# MicroRNA-1291 targets the FOXA2-AGR2 pathway to suppress pancreatic cancer cell proliferation and tumorigenesis

**DOI:** 10.18632/oncotarget.9999

**Published:** 2016-06-14

**Authors:** Mei-Juan Tu, Yu-Zhuo Pan, Jing-Xin Qiu, Edward J. Kim, Ai-Ming Yu

**Affiliations:** ^1^ Department of Biochemistry and Molecular Medicine, UC Davis School of Medicine, Sacramento, CA 95817, USA; ^2^ Department of Pharmaceutical Sciences, SUNY-Buffalo, Buffalo, NY 14214, USA; ^3^ Department of Pathology, Roswell Park Cancer Institute, Buffalo, NY 14263, USA; ^4^ Division of Hematology and Oncology, UC Davis Comprehensive Cancer Center, Sacramento, CA 95817, USA

**Keywords:** microRNA, miR-1291, AGR2, FOXA2, pancreatic cancer

## Abstract

Pancreatic cancer is the fourth leading cause of cancer death in the United States. Better understanding of pancreatic cancer biology may help identify new oncotargets towards more effective therapies. This study investigated the mechanistic actions of microRNA-1291 (miR-1291) in the suppression of pancreatic tumorigenesis. Our data showed that miR-1291 was downregulated in a set of clinical pancreatic carcinoma specimens and human pancreatic cancer cell lines. Restoration of miR-1291 expression inhibited pancreatic cancer cell proliferation, which was associated with cell cycle arrest and enhanced apoptosis. Furthermore, miR-1291 sharply suppressed the tumorigenicity of PANC-1 cells in mouse models. A proteomic profiling study revealed 32 proteins altered over 2-fold in miR-1291-expressing PANC-1 cells that could be assembled into multiple critical pathways for cancer. Among them anterior gradient 2 (AGR2) was reduced to the greatest degree. Through computational and experimental studies we further identified that forkhead box protein A2 (FOXA2), a transcription factor governing AGR2 expression, was a direct target of miR-1291. These results connect miR-1291 to the FOXA2-AGR2 regulatory pathway in the suppression of pancreatic cancer cell proliferation and tumorigenesis, providing new insight into the development of miRNA-based therapy to combat pancreatic cancer.

## INTRODUCTION

Pancreatic adenocarcinoma is one of the most lethal malignancies in the world. It is the fourth most common cause of cancer-related deaths with a 5-year survival rate of 6%, and around 40,000 patients die from pancreatic cancer each year in the United States [1]. Pancreatic ductal adenocarcinoma (PDAC) is by far the most common type of pancreatic cancer and accounts for more than 85% of the different histologic subtypes of pancreatic cancer [2–4]. The mortality rate for pancreatic cancer remains high due to multiple factors including an inherently aggressive metastatic nature with early dissemination, a lack of reliable screening methods to make early diagnosis, and most importantly the lack of more effective therapies [1, 5–7]. One potential curative treatment for pancreatic cancer is surgical resection. However, less than 15% of patients have disease amenable to surgery at the time of diagnosis [8, 9] and median survival after surgical resection and adjuvant chemotherapy is only about 20–24 months with 5 year survival rate of around 20% [10, 11]. Although our knowledge of pancreatic cancer biology is increasing, the causes of pancreatic cancer remain largely unknown and more effective therapies still await discovery and development. Therefore, identifying new targets and therapeutic strategy for patients with pancreatic carcinoma continues to be an urgent need.

MicroRNAs (miRs or miRNAs) are genome-derived noncoding RNA (ncRNA) molecules that govern target gene expression in cells in a sequence-specific manner [12–14]. Many miRNAs are revealed to play critical roles in the control of cancer cellular processes including proliferation, cell cycle, apoptosis, invasion, tumorigenesis and metastasis, which opens new avenues to develop miRNA-based therapies. As an example, miR-34a replacement therapy has entered Phase I clinical trial for the treatment of unresectable primary liver cancer [15, 16]. There are also some studies demonstrating that a number of miRNAs are aberrantly expressed in pancreatic cancer cells/tissues and involved in the regulation of many pancreatic cancer related genes [17–27]. Therefore, investigation of novel miRNA regulatory pathways may offer insights into identification of new pancreatic oncotargets and development of new therapeutics [7, 28, 29].

Our recent studies have identified that microRNA-1291 (miR-1291), a less studied miRNA that is generated from small nucleolar RNA H/ACA box 34 (SNORA34) in pancreatic cancer PANC-1 cells, is able to increase intracellular drug accumulation and chemosensitivity through targeting of efflux transporter namely multidrug resistance-associated protein 1 (MRP1/ABCC1) [30, 31]. We have also demonstrated that miR-1291 modulates the metabolome of PANC-1 cells and reduces cell migration and invasion capacity [32]. Other studies showed that miR-1291 is downregulated in clinical renal cell carcinoma specimens [33], and gain of miR-1291 function inhibits the proliferation, migration and invasion of renal carcinoma A498 and 786-O cells [34].

The objective of the current study was to delineate the role of miR-1291 in pancreatic cancer. We first showed that miR-1291 was significantly downregulated in patient PDAC tissues, and restoration of miR-1291 expression/function inhibited pancreatic cancer cell proliferation *in vitro* through induction of G2/M cell cycle arrest and enhancement of apoptosis. Then we demonstrated miR-1291 sharply suppressed tumorigenicity of PANC-1 cells in xenograft mouse models *in vivo*. Proteomic profiling study revealed a set of proteins altered by miR-1291 that formed a network of interactions in the control of critical cancer properties. The most significantly downregulated protein was anterior gradient 2 (AGR2), which was conversely overexpressed in PDAC tissues. In addition, the transcription regulator of AGR2, forkhead box protein A2 (FOXA2) [35–37], was identified as a direct target of miR-1291. Together, our results link miR-1291 to FOXA2-AGR2 regulatory pathway in the suppression of pancreatic tumorigenesis. These findings improve the understanding of pancreatic miRNA oncotargets and provide insight that supports the development of miR-1291-based therapy for the treatment of pancreatic cancer.

## RESULTS

### MiR-1291 expression is downregulated in human pancreatic cancer cell lines and patient tumor specimens

**Figure 1:**
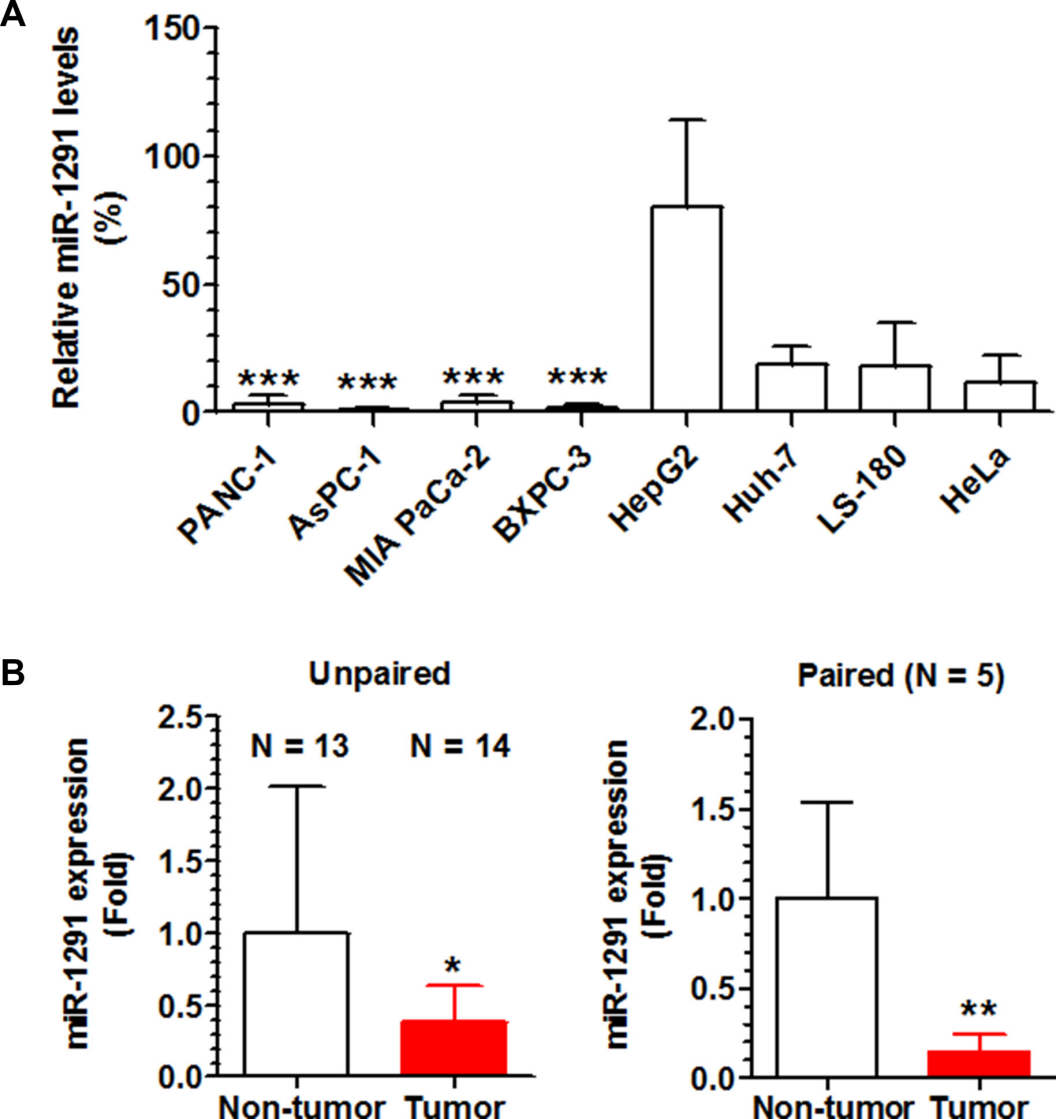
miR-1291 expression levels are lower in human pancreatic cancer cell lines and patient PDAC tissues (**A**) The expression levels of miR-1291 were remarkably lower in human pancreatic cancer cell lines than other cell lines. ***P < 0.001, compared to HepG2 cells. Values are mean ± SD (*N* = 3). (**B**) The average expression level of miR-1291 was about 60% lower in PDAC tissues than unpaired non-tumor tissues, and over 6-fold lower than paired peripheral non-tumor tissues. *P < 0.05, and **P < 0.01; values are mean ± SD.

We first measured miR-1291 levels in human pancreatic cancer cell lines (PANC-1, AsPC-1, BXPC-3, and MIA PaCa-2) and compared to non-pancreatic cell lines including the hepatic cancer cell lines HepG2 and Huh7, colon carcinoma LS-180 cells, and cervical carcinoma Hela cells. The data showed that miR-1291 levels were remarkably lower in each of the four pancreatic cancer cell lines compared to the other cancer cell lines (Figure 1A). We then examined miR-1291 expression levels in patient PDAC tissues in comparison to normal pancreatic tissues. As shown in Figure 1B, miR-1291 levels were about 60% lower in PDAC tissues than unpaired non-tumor tissues. A more striking difference was seen in the five sets of paired specimens in which miR-1291 levels were 6-fold lower in tumor samples than their paired normal pancreatic tissues (Figure 1B). The decreased expression of miR-1291 found in human pancreatic cancer cell lines and PDAC tissues suggest that miR-1291 might be related to pancreatic tumorigenesis.

### Restoration of miR-1291 expression reduces human pancreatic cancer cell proliferation by inducing G2/M cell cycle arrest and enhancing apoptosis

**Figure 2:**
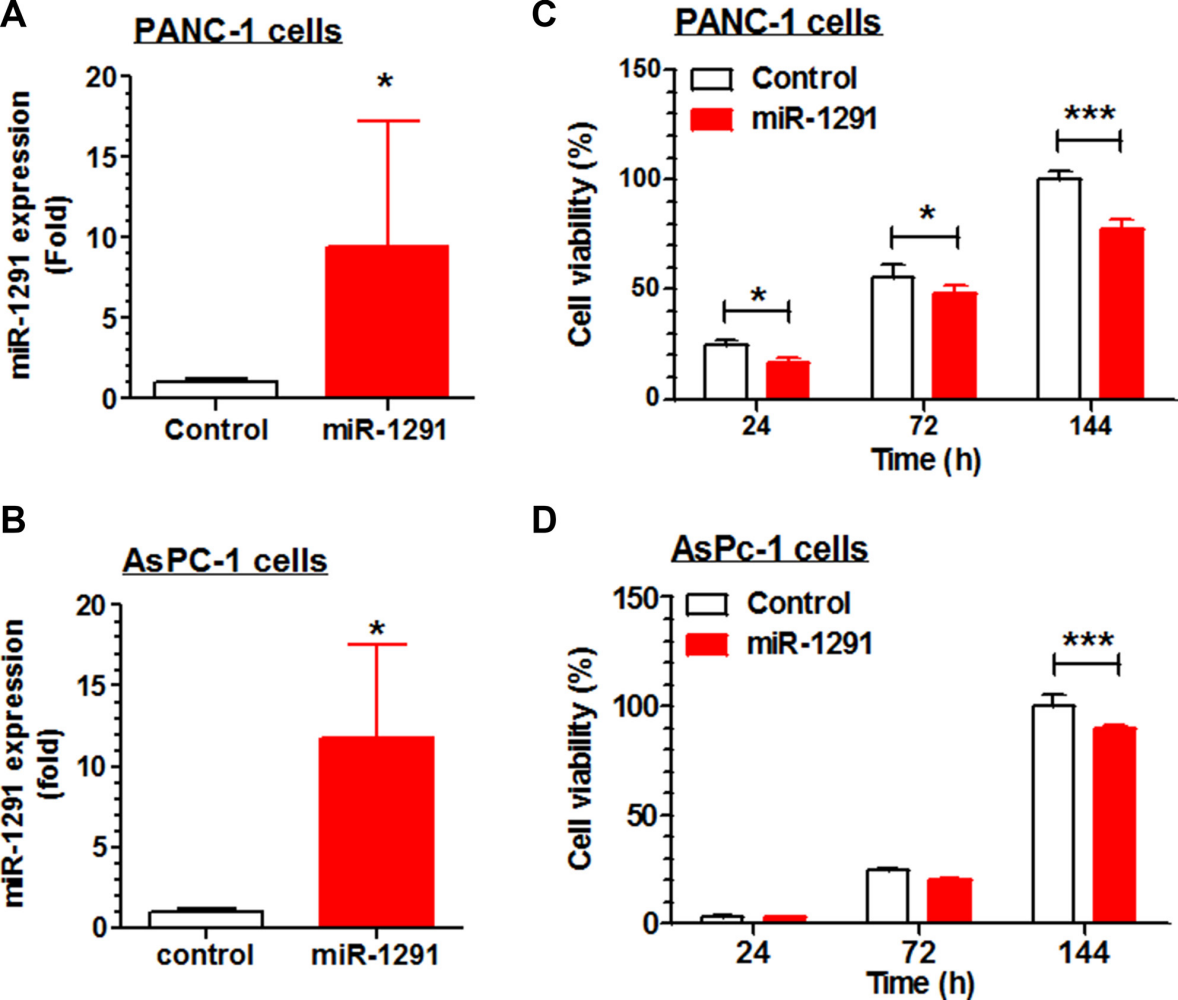
Restoration of miR-1291 expression suppresses the proliferation of PANC-1 and AsPC-1 cells (**A, B**) miR-1291 expression levels were about 9- and 12-fold higher in miR-1291 stably transfected PANC-1 and AsPc-1 cells, respectively, as compared to corresponding control cells transfected with empty vectors. (**C, D**) Cell proliferation capacity was significantly reduced in the miR-1291-expressing PANC-1 and AsPC-1 cells, as determined by MTT assays. Viability of control cells at the last time point was set as 100%. Values are mean ± SD (*N* =3). ***P < 0.001, *P < 0.05, compared to control cells.

To delineate the potential role of miR-1291 in pancreatic cancer, we first investigate the effects of restoration of miR-1291 expression/function on pancreatic cancer cell proliferation. AsPC-1 and PANC-1 cells transiently transfected with miR-1291 expression plasmid exhibited about 50% lower viabilities, compared to cells transfected with empty vectors (data not shown). We thus generated stable miR-1291-expressing AsPC-1 and PANC-1 cells to explore potential mechanisms. Compared to corresponding controls, miR-1291-expressing PANC-1 and AsPC-1 cells showed approximately 9- (Figure 2A) and 12-fold (Figure 2B) higher miR-1291 levels, which resulted in a significantly lower cell proliferation capacity (Figure 2C and 2D). Since PANC-1 cells were more sensitive to miR-1291 than AsPC-1 cells, PANC-1 cell lines were utilized for further studies.

**Figure 3:**
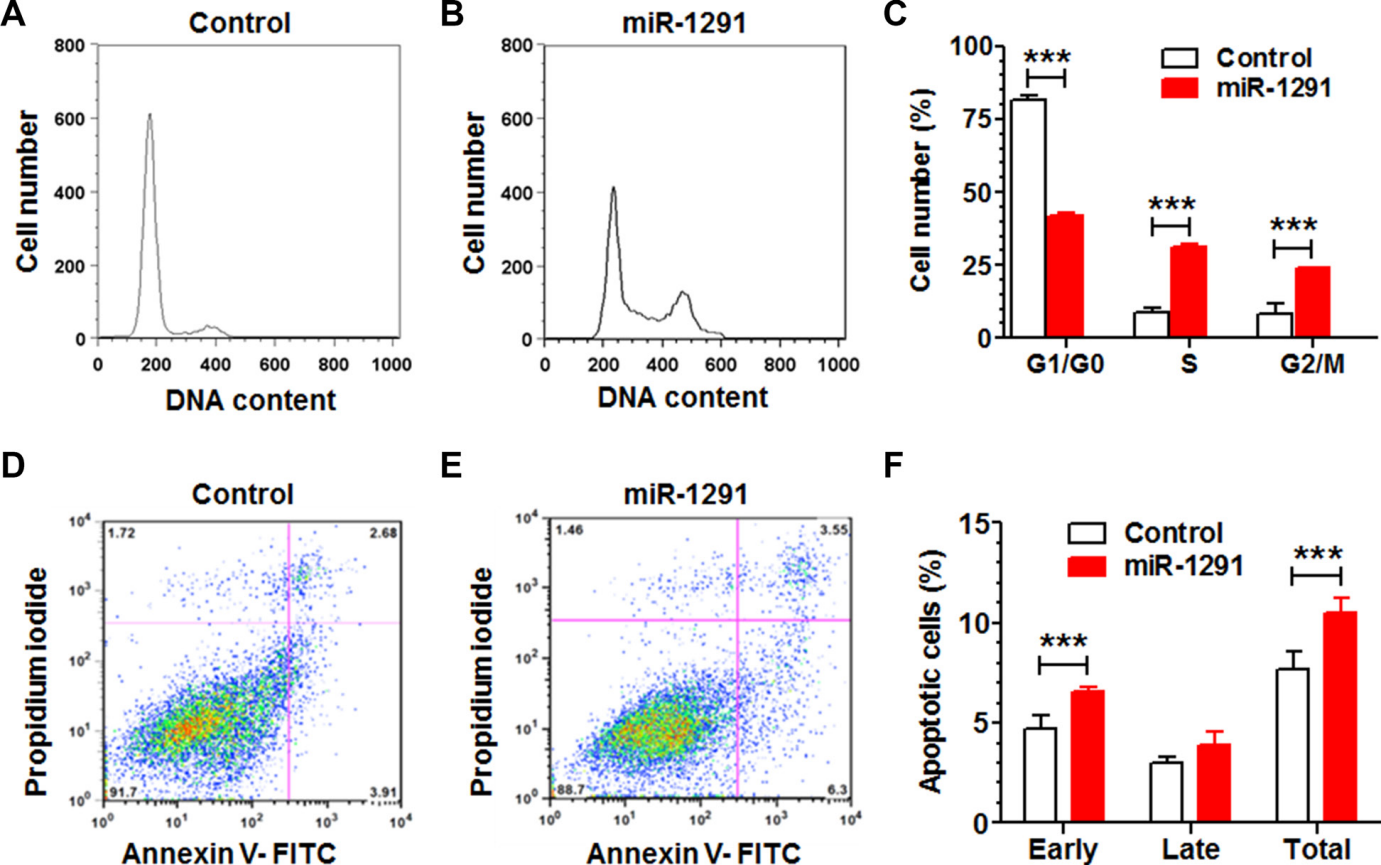
Reintroduction of miR-1291 into PANC-1 cells induces a G2/M cell cycle arrest and an enhanced apoptosis (**A, B**) Comparison of flow cytometry histograms of control and miR-1291-expressing PANC-1 cells stained with propidium iodide, and (**C**) the percentage of cells at various phases (G1/G0, S and G2/M). (**D, E**) Comparison of flow cytometry histograms of control and miR-1291-expressing cells stained with Annexin V/propidium iodide, and (**F**) the percentage of apoptotic cells. Values are mean ± SD (*N* = 3). ***P < 0.001,*P < 0.05, compared to corresponding controls.

To assess whether the inhibition of pancreatic cancer cell proliferation by miR-1291 involves mechanistic changes of cell cycle and apoptosis, we measured cell cycle (Figure 3A–3C) and apoptotic (Figure 3D–3F) profiles through flow cytometric analyses of propidium iodide and Annexin V/propidium iodide stained cells, respectively. Our data showed that restoration of miR-1291 expression led to a 2-fold increase of PANC-1 cells in G2/M phase, which was accompanied by a significant reduction of cells in G1 phase and increase of cells in S phase (Figure 3A–3C). In addition, the fraction of early apoptotic cells was increased by 40% in miR-1291-expressing PANC-1 cells (Figure 3D–3F). Together, these results demonstrate that miR-1291 inhibits pancreatic cancer cell proliferation (Figure 2) via the induction of G2/M cell cycle arrest and enhancement of early apoptosis (Figure 3).

### MiR-1291 suppresses the tumorigenicity of human pancreatic cancer cells in mouse models

**Figure 4:**
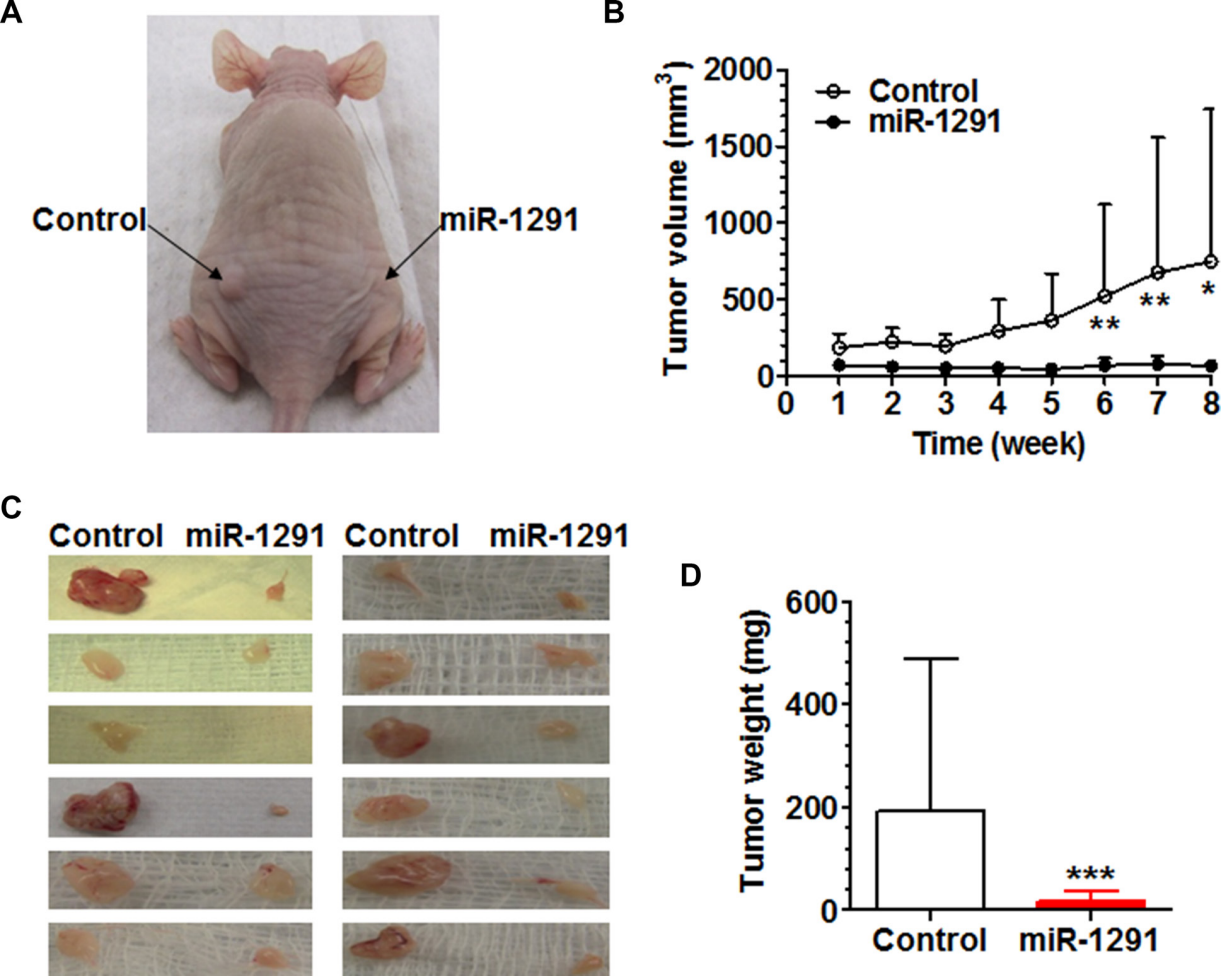
miR-1291 suppresses the tumorigenicity of PANC-1 cells in xenograft tumor mouse models (**A**) Representative picture of mouse inoculated with control and miR-1291-expressing PANC-1 cells. (**B**) Growth of xenograft tumors from miR-1291-expressing PANC-1 cells was significantly (*P* < 0.001, two-way ANOVA) slower than the control cells. **P < 0.01 and *P < 0.05, compared to the same time points. (**C**) Visual comparison of xenograft tumors excised from individual mice at week 8 after inoculation. (**D**) Xenograft tumors derived from miR-1291-expressing PANC-1 cells was significantly (***P < 0.001) lighter than the control cells. Values are mean ± SD (*N* = 12).

To further define the impact of miR-1291 on the tumorigenesis of pancreatic cancer cells, miR-1291-expressing and control PANC-1 cells were injected subcutaneously into the right and left side of the dorsum of nude mouse, respectively, and outgrowth of xenograft tumors was monitored. The data revealed that growth of PANC-1 xenograft tumors in the same mouse was remarkably and significantly suppressed by miR-1291 (Figure 4A and 4B). In addition, xenograft tumors derived from miR-1291-expressing PANC-1 cells were much smaller (Figure 4C) and over 10-fold lighter (Figure 4D) than the paired specimens generated from control PANC-1 cells. These results indicate that miR-1291 is able to suppress the tumorigenicity of pancreatic cancer cells in xenograft mouse models.

### MiR-1291 modulates the expression of many proteins in PANC-1 cells that are assembled into a network of tumor regulatory pathways

**Figure 5:**
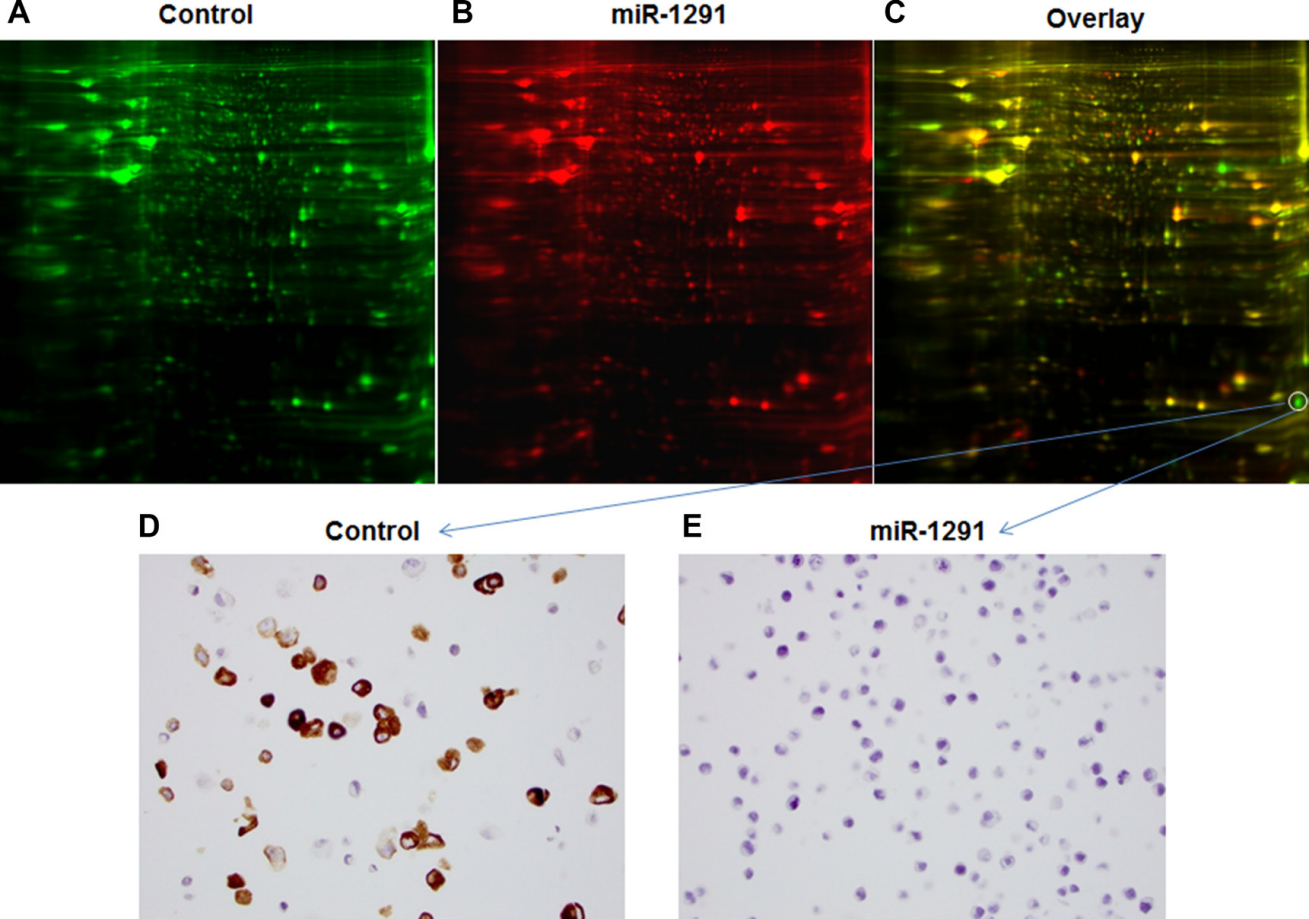
Difference in global protein expression profiles between miR-1291-expressing and control PANC-1 cells (**A–C**) 2D-DIGE images of proteins in the control and miR-1291-expression PANC-1 cells labeled with green and red fluorescent dye, respectively, and the overlaid graph indicating the difference in the abundance of proteins. The protein downregulated in miR-1291-expressing PANC-1 cells to the greatest degree was identified as AGR2. (**D, E**) Immunocytochemistry analysis confirmed the sharp downregulation of AGR2 (brown staining) in miR-1291-expressing PANC-1 cells (400 ×).

**Figure 6:**
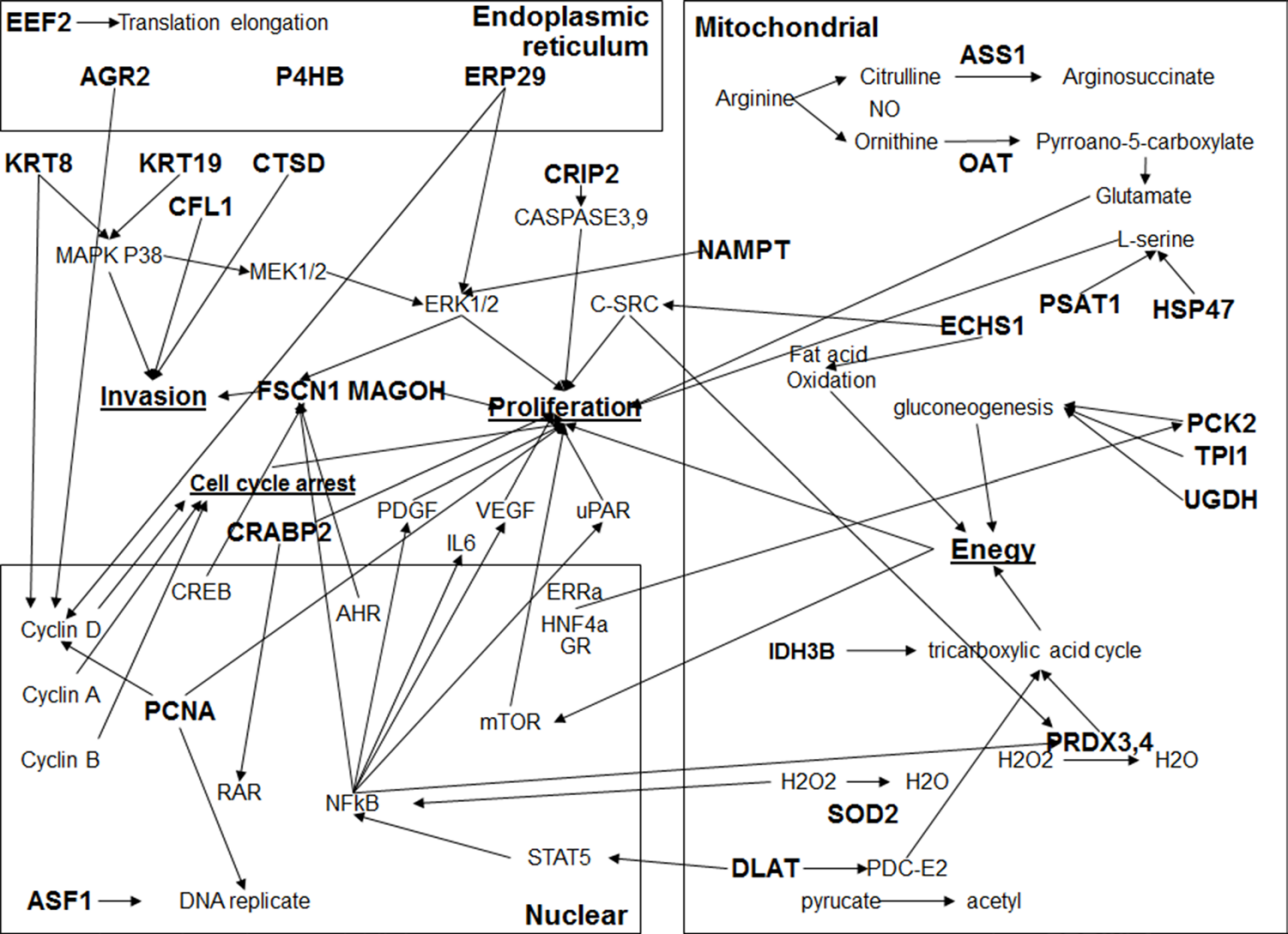
The network of interactions among miR-1291-modualted proteins Network of interactions was constructed using IPA software. The input was all miR-1291-altered proteins (in bold) in PANC-1 cells identified by 2D-DIGE, MALDI-TOF and MS/MS proteomic profiling study.

To understand possible signaling pathways underlying miR-1291-controlled suppression of pancreatic tumorigenesis, an unbiased comprehensive proteomic profiling study was conducted to determine global protein expression altered by miR-1291 (Figure 5). Our study using two-dimensional difference gel electrophoresis (2D-DIGE), matrix-assisted laser desorption/ionization (MALDI)-time of flight (TOF) and MS/MS analyses revealed 19 downregulated proteins and 13 upregulated proteins whose levels were altered over 2-fold in miR-1291-expressing PANC-1 cells (Table 1, Figure 5A–5C). The pro-oncogenic protein AGR2 was revealed to be the one downregulated to the greatest degree (over 9-fold; Table 1), which was further confirmed by immunoblot (data not shown) and immunocytochemistry (Figure 5D and 5E). In addition, pathway analysis demonstrated that miR-1291-altered proteins formed a network of interactions in the modulation of many cancer cellular processes including cell proliferation, cell cycle arrest, invasion, endoplasmic reticulum stress, and energy metabolism (Figure 6). These results suggest that miR-1291 modulates a network of important tumor-regulatory pathways in pancreatic cancer cells.

**Table 1:** Proteins differentially expressed in miR-1291-expressing and control PANC-1 cells, which were identified by 2D-DIGE, MALDI-TOF and MS/MS proteomic profiling study

Fold of change (miR-1291/control)	Top Ranked Protein Name (Species)	Gene
−9.49	Anterior gradient homolog 2 [Homo sapiens]	AGR2
−7.95	Argininosuccinate synthase [Homo sapiens]	ASS1
−4.03	Chain C, Structure Of The H3-H4 Chaperone Asf1Bound To Histones H3 And H4	ASF1
−2.77	Ornithine aminotransferase, mitochondrial isoform 1 precursor [Homo sapiens]	OAT
−2.50	Keratin, type II cytoskeletal 8 [Homo sapiens]	KRT8
−2.45	Phosphoenolpyruvate carboxykinase 2 (mitochondrial) [Homo sapiens]	PCK2
−2.44	Chain A, The Crystal Structure Of Human Enoyl-Coenzyme A (Coa) Hydratase Short Chain 1, Echs1	ECHS1
−2.43	Phosphoserine aminotransferase isoform 1 [Homo sapiens]	PSAT1
−2.41	Dihydrolipoamide acetyltransferase [Homo sapiens]	DLAT
−2.39	Peroxiredoxin 3, isoform CRA_a [Homo sapiens]	PRDX3
−2.39	Cysteine-rich protein 2 [Homo sapiens]	CRIP2
−2.32	Chain C, Human Pcna	PCNA
−2.20	Fascin homolog 1, actin-bundling protein (Strongylocentrotus purpuratus), isoform CRA a [Homo sapiens]	FSCN1
−2.19	Serpin H1 precursor [Homo sapiens]	HSP47
−2.15	Protein disulfide-isomerase precursor [Homo sapiens]	P4HB
−2.15	Chain A, Crystal Structure Of The Protein-Disulfide Isomerase Related Chaperone Erp29	ERP29
−2.15	Triosephosphate isomerase isoform 2 [Homo sapiens]	TPI1
−2.07	Peroxiredoxin-4 [Homo sapiens]	PRDX4
−2.00	Isocitrate dehydrogenase [NAD] subunit beta, mitochondrial isoform a precursor [Homo sapiens]	IDH3B
2.39	Superoxide dismutase [Mn], mitochondrial isoform A precursor [Homo sapiens]	SOD2
3.49	Nicotinamide phosphoribosyltransferase precursor [Homo sapiens]	NAMPT
3.69	Chain B, Crystal Structures Of Native And Inhibited Forms Of Human Cathepsin D: Implications For Ly	CTSD
3.92	Chain A, Cellular Retinoic Acid Binding Protein Ii In Complex With A Synthetic Retinoic Acid (Ro-13)	CRABP2
5.10	Cofilin-1 [Homo sapiens]	CFL1
5.31	Elongation factor 2 [Homo sapiens]	EEF2
5.53	Protein mago nashi homolog [Homo sapiens]	MAGOH
6.58	UDP-glucose 6-dehydrogenase isoform 1 [Homo sapiens]	UGDH
6.60	Protein S100-A13 [Homo sapiens]	S100A13
6.97	Destrin isoform a [Homo sapiens]	DSTN
7.20	UDP-glucose 6-dehydrogenase isoform 1 [Homo sapiens]	UGDH
12.4	Keratin, type I cytoskeletal 19 [Homo sapiens]	KRT19
26.4	Chain A, Cellular Retinoic Acid Binding Protein Ii In Complex With A Synthetic Retinoic Acid (Ro-13)	CRABP2

The cut-off was 2-fold change.

### MiR-1291 is linked to FOXA2-AGR2 pathway in pancreatic cancer

**Figure 7:**
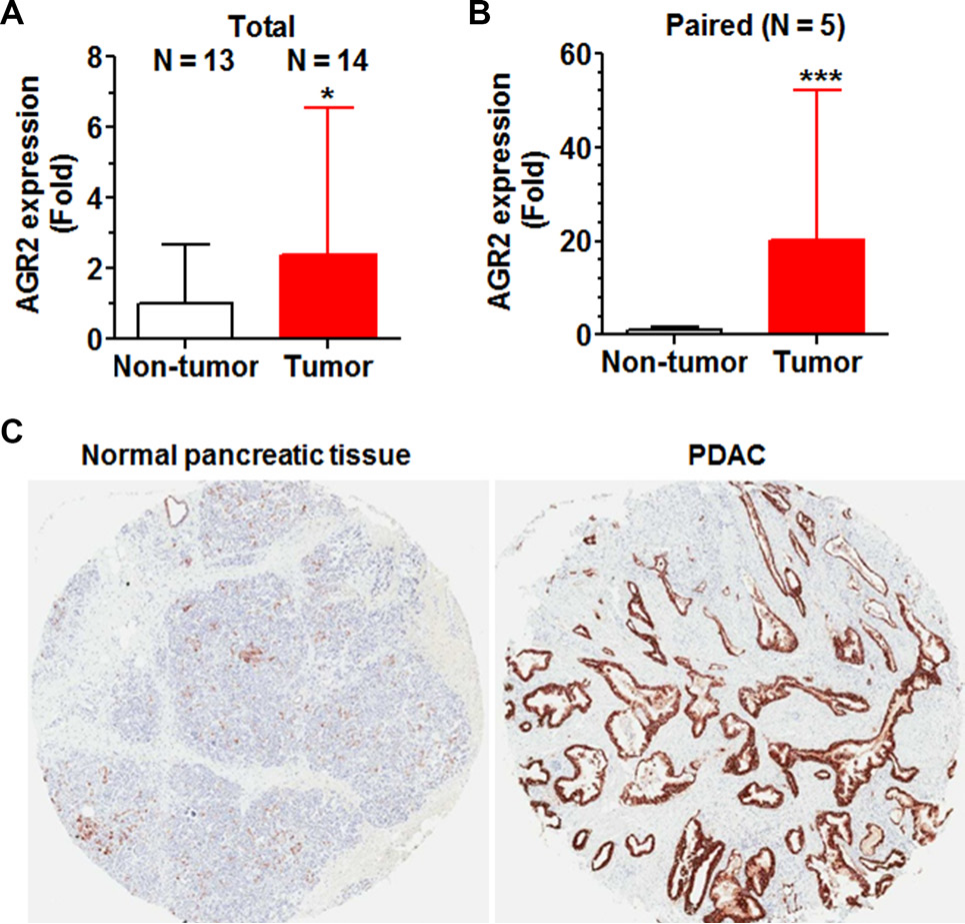
AGR2 is overexpressed in patient PDAC tissues, which is in contrast to the reduced expression of miR-1291 (Figure 1B) (**A, B**) AGR2 mRNA levels were significantly higher in pancreatic tumor tissues than non-tumor tissues, as determined by selective qPCR analyses. (**C**) AGR2 protein (brown stains) levels were much higher in patient PDAC than adjunct normal pancreatic tissues, as demonstrated by immunohistochemistry study.

Because AGR2 was down-regulated to the greatest extent in miR-1291-expressing PANC-1 cells and AGR2 is a known proto-oncogene in the control of cancer cell proliferation, invasion and transformation [38–41], the clinical PDAC samples (Figure 1B) were thus employed to critically evaluate the relationship between miR-1291 and AGR2. RT-qPCR analyses showed that AGR2 mRNA levels were over 2-fold and 20-fold higher in pancreatic tumor tissues than unpaired and paired non-tumor tissues (Figure 7A and 7B). Further immunohistochemistry analyses revealed a much greater level of AGR2 protein expression in PDAC tissues than adjunct normal pancreatic tissues (Figure 7C). The overexpression of AGR2 (Figure 7) and downregulation of miR-1291 (Figure 1B) in pancreatic carcinoma indicates an inverse relationship between miR-1291 and AGR2 in pancreatic cancer.

**Figure 8:**
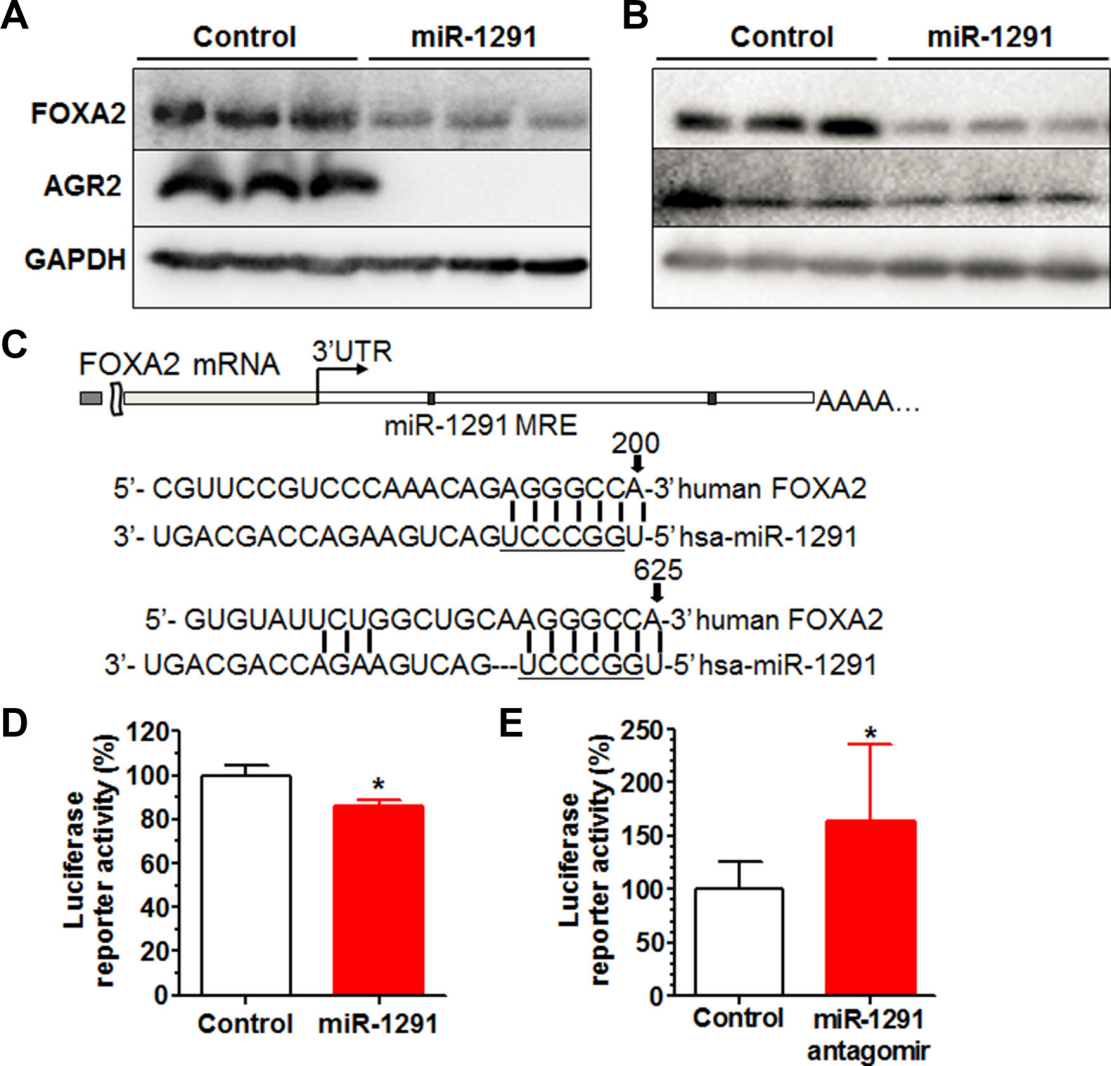
miR-1291 modulates AGR2 expression through targeting of FOXA2, a known transcriptional regulator of AGR2 (**A, B**) Western blot analysis showed that both FOXA2 and AGR2 protein levels were much lower in miR-1291-expressing PANC-1 (A) and AsPC-1 (B) cells, as compared to corresponding control cells. GAPDH was used as a loading control. (**C**) Computational analysis revealed two putative MRE sites for miR-1291 within the 3′UTR of FOXA2. Underlined is the seed sequence of miR-1291. (**D, E**) FOXA2 3′UTR luciferase reporter activities were decreased in PANC-1 cells (D) and increased in HepG2 cells (E) after the transfection with miR-1291 expression plasmid and antagomir, respectively. *P < 0.05, compared to the control group. Values are mean ± SD (*N* = 9).

Our efforts were thus directed to understand how miR-1291 might regulate AGR2 expression in pancreatic cancer cells. While bioinformatics analyses indicated that AGR2 did not appear to be a direct target of miR-1291, FOXA2, the transcription regulator of AGR2 [35–37], was predicted to be a potential target of miR-1291. Indeed the lower level of AGR2 protein expression in miR-1291-expressing PANC-1 (Figure 8A) and AsPC-1 (Figure 8B) cells was associated with a reduced level of FOXA2 protein expression. To evaluate the action of miR-1291 on the 3′UTR of FOXA2 consisting of two putative miRNA response elements (MREs) for miR-1291 (Figure 7C), we further constructed a FOXA2 3′UTR-luciferase reporter plasmid. Consequent luciferase reporter assays showed that gain and loss of miR-1291 expression/function was able to significantly reduce and increase FOXA2 3′UTR luciferase activities, respectively (Figure 8D and 8E), supporting that FOXA2 is a direct target of miR-1291. These results, along with the previous findings on the regulation of AGR2 by FOXA2 [35–37], demonstrate that miR-1291 modulates AGR2 expression via targeting of its transcription factor FOXA2.

## DISCUSSION

There are increasing number of studies demonstrating aberrant expression of miRNAs in a multitude of cancers and supporting the contribution of regulatory miRNAs to the initiation, progression, and metastasis of cancers. The current study revealed a significantly lower miR-1291 level in pancreatic tumor specimens as well as pancreatic cancer cell lines. Our data also demonstrated that re-introduction of miR-1291 to pancreatic cancer cells remarkably suppressed tumorigenesis in xenograft mouse models, which might be attributable to the inhibition of cell proliferation, induction of G2/M cell cycle arrest, and enhancement of apoptosis. Furthermore, we identified a set of proteins altered in PANC-1 cells by miR-1291 that were assembled into critical tumor regulatory pathways. In addition, FOXA2 was revealed as a direct target for miR-1291, which connected miR-1291 to the established FOXA2-AGR2 regulatory pathway [35–37] in the control of pancreatic cancer cell properties.

MiR-1291 belongs to a special group of miRNAs that are derived from small nucleolar RNAs [30]. Recent studies have showed that miR-1291 level is lower in renal cell carcinoma specimens [33] and esophageal squamous cell carcinoma [42], and restoration of miR-1291 suppresses renal and esophageal squamous cancer cell proliferation, migration and invasion [34, 42]. The present study is the first to find that miR-1291 is significantly downregulated in patient pancreatic carcinoma tissues and pancreatic cancer cell lines. Consistent with our recent findings on the antiproliferative activity of miR-1291 [31], this study further demonstrated that restoration of miR-1291 expression/function significantly inhibited the growth of human pancreatic cancer cells, which was associated with an accumulation of cells in G2/M phase and larger fractions of apoptotic cells. In addition, miR-1291 was revealed to largely suppress the tumorigenesis of PANC-1 cells, in which the same mouse was inoculated with both miR-1291-expressing and control PANC-1 cells. This study design is distinguished from our recent report that involves the use of different mice for xenograft tumor study [32], and current tumor mouse model not only allows the reduction of number of experimental animals for research but also minimizes interindividual variability and other confounding factors. Nevertheless, consistent results are obtained from present and previous studies [31, 32, 34, 42], indicating that miR-1291 acts as a tumor suppressor in renal, esophageal squamous, and pancreatic cancer cells.

Several targets identified for miR-1291 thus far include the efflux transporter ABCC1 underlying multidrug resistance [30], the endoplasmic reticulum stress sensor *IRE1α* and consequently glypican-3 (GPC3) implied in liver carcinogenesis [43, 44], glucose transporter protein type 1 (GLUT1/SLC2A1) critical for cell metabolism [34], and mucin 1 (MUC1) making up mucus [42]. Our recent metabolomics study have revealed nicotinamide *N*-methyltransferase (NNMT), an enzyme participating in nicotinate and nicotinamide metabolism, as a biomarker for miR-1291-altered pancreatic cancer cell metabolome [32]. The proteomics analysis in current study successfully identified a set of miR-1291-altered proteins that were assembled into multiple pathways important for cancer cellular processes including cell proliferation, cell cycle and apoptosis that were investigated in present study, as well as other cell functions such as fatty acid metabolism and gluconeogenesis. Although the connection between FOXA2-AGR2 signaling and nicotinate and nicotinamide metabolism is unknown, the disruption of fatty acid metabolism disclosed by metabolomics study [32] may be explained by the change of ECHS1 identified by present proteomics study. Most importantly, this study found that a series of cell metabolism-related proteins such as ASS1, OAT, UGDH, and PCK2 were significantly altered in miR-1291 expressing cells. Indeed, change of NNMT status was shown to alter the sensitivity of PANC-1 cells to glucose deprivation and rapamycin as well as glycolytic inhibitor 2-deoxyglucose [45]. Rather, precise relationships between miR-1291 and these proteins in the control of specific cancer cellular processes need further investigation.

Oncogenic AGR2 was identified as the most significantly downregulated protein in miR-1291-expressing PANC-1 cells with lower capacity in proliferation and tumorigenesis, consistent with previous findings on the critical role of AGR2 in pancreatic cancer [38, 40]. Indeed AGR2 is overexpressed throughout the progression to pancreatic cancer and has been shown to facilitate the initiation, progression, and dissemination of pancreatic cancer [38, 40, 46]. In addition, upregulation of AGR2 promotes the expression of MUC1 [46], which acts as an important regulator of the metabolism of pancreatic cancer cells [47]. A sharp reduction of AGR2 (current study) and significant change of cell metabolome [32] are unified for miR-1291-expression PANC-1 cells, suggesting that miR-1291 may be implicated in the metabolism of pancreatic cancer cells. Consistent with previous studies, our findings support the notion that AGR2 represents a druggable oncotarget for the development of new therapies to treat notoriously lethal pancreatic cancer.

Although many genes encoding those altered proteins in miR-1291-expressing PANC-1 cells do not seem to be directly regulated by miR-1291, the present study identified FOXA2 as a direct target for miR-1291. Luciferase assays supported the interaction of miR-1291 with FOXA2 3′UTR consisting of miR-1291 MRE sites predicted by bioinformatic analysis. Furthermore, consistent with the results obtained from miR-1291-expressing pancreatic cancer cells (this study), both FOXA2 and AGR2 protein levels were reduced in cells after transient transfection with miR-1291 expression plasmids (data not shown) or bioengineered miR-1291 agent [31]. While further biological experiments are necessary to validate FOXA2 as a direct target of miR-1291, this study has firmly established the impact of miR-1291 on FOXA2. Most importantly, FOXA2 is a member of the fork head transcription factor family that binds to the promoter region of AGR2 and activates the transcription of AGR2 [35–37]. FOXA2 is also essential for cell differentiation, metabolism, and maturation including pancreas development and epithelial-to-mesenchymal transition [48, 49]. Therefore, current study unveiled the connection between miR-1291 and FOXA2-AGR2, namely miR-1291-FOXA2-AGR2 pathway, in the modulation of pancreatic cancer cellular processes.

It is possible that other genes and pathways might be also involved in miR-1291-caused downregulation of AGR2 (as well as changes of other proteins listed in Table 1) in suppressing the proliferation and tumorigenesis of pancreatic cancer cells. Actually AGR2 protein levels were reduced to different degrees in miR-1291-expressing PANC-1 and AsPC-1 cells, highlighting intrinsic difference in the regulatory pathways beyond miR-1291-FOXA2-AGR2 between the two cells lines. In addition, the extent of decrease in AGR2 protein levels was greater than that of FOXA2 in miR-1291-expressing PANC-1 cells, suggesting the presence of other pathways and an accumulative effect on the suppression of AGR2 expression. Besides FOXA2, several other cancer-related genes such as protein kinase B AKT2 [50–52] and methyl-CpG binding protein 2 (MeCP2) [53–55] were also identified as putative targets for miR-1291 by computational analysis. The protein levels of AKT2 and MeCP2 were indeed reduced in miR-129-expressing PANC-1 cells (unpublished data) or MCF-7 cells transiently transfected with miR-1291 agent [31]. Therefore, further studies on additional targets of miR-1291 are necessary to provide an improved mechanistic understanding of miR-1291 pathways in the regulation of pancreatic cancer processes.

In conclusion, the present study revealed a miR-1291-FOXA2-AGR2 signaling pathway behind miR-1291-controlled suppression of pancreatic tumorigenesis. Our results demonstrated that miR-1291 was significantly downregulated in pancreatic cancer specimens, which was in contrast to the overexpression of oncogenic AGR2. Furthermore, restoration of miR-1291 expression inhibited pancreatic cancer cell proliferation that was attributed to the induction of G2/M cell cycle arrest and apoptosis. These findings improve our understanding of pancreatic cancer and critical regulatory pathways which provide insight into the development of miR-1291-based therapeutic strategy.

## MATERIALS AND METHODS

### De-identified patient samples

The de-identified pancreatic cancer patient samples and non-tumor tissues were obtained from the Pathology Resource Network of Roswell Park Cancer Institute (RPCI). All samples were processed according to the standard pathology procedures at the RPCI including quality control and verification by pathologist. A total of 22 tissue specimens from non-Spanish, White patients were used for this study, among which 7 donors are females and 15 donors are males. The ages of patients were between 31 and 84 years at enrollments, with a mean value of 63.3 and standard deviation (SD) of 13.6. Samples were subjected to biochemical and immunohistochemistry analyses under the protocol NHR 022611 approved by the Office of Research Subject Protection at RPCI.

### Plasmids

The coding sequence of FOXA2 (NM_021784.4) 3′UTR segment (0–830 bp from stop codon) consisting of miR-1291 MRE sites that were predicted by TargetScan (http://www.targetscan.org/) was amplified from human genome by PCR using gene specific primers, 5′-CCG CTC GAG GGG GTG TAC TCC CGG CCC ATT ATG AAC TCC TCT-3′ (forward) and 5′-TTG CGG CCG CGG GCC AAA ATA AAA TAC AAC CTG CAA CCA GAC A-3′ (reverse), and cloned downstream of *Renilla* luciferase gene within psiCHECK-2 vector after digested with XhoI and NotI. The FOXA2 3′UTR-luciferase reporter plasmid was confirmed by direct DNA sequencing and named as psiCHECK-FOXA2–3 ′UTR. The miR-1291 expression plasmid and the control empty vector were described recently [30].

### Cell culture

Human pancreatic cancer cell lines PANC-1, AsPC-1, MIA PaCa-2, BXPC-3, liver cancer HepG2 cells, colon carcinoma LS-180 cells, and cervical carcinoma HeLa cells were purchased from ATCC (Manassas, VA), and human hepatocellular carcinoma Huh-7 cells were bought from Riken Cell Bank (Wako, Saitama, Japan). All cells were maintained in Dulbecco's Modified Eagle Medium (DMEM) or Eagle's Minimum Essential Medium (EMEM) containing 10% FBS, 100 U/ml of penicillin sodium, and 100 μg/ml of streptomycin sulfate at 37°C in a humidified atmosphere of 5% CO_2_. The miR-1291-expressing and control PANC-1 cells were established recently in our lab [30]. AsPC-1 cells stably transfected with miR-1291 expression plasmid and empty control vector were developed in the same manner.

### Cell viability

Cells were seeded in 24-well plates at a density of 4 × 10^3^ (AsPC-1 cells) or 5 × 10^3^ (PANC-1 cells), and cell viability was evaluated at various time points (24, 72, and 144 h) using MTT assay, as described [56].

### Cell cycle and apoptosis analyses

Percentages of apoptotic cells and cell cycle phases were determined using a FACSCalibur flow cytometer (BD Biosciences, San Jose, CA) after the cells were stained with Annexin-V FITC Apoptosis Kit (Invitrogen, Carlsbad, CA) and propidium iodide/RNase (Sigma-Aldrich, St. Louis, MO), respectively, as we described recently [56]. All experiments were carried out in triplicate with separate cultures and all data were analyzed with Flowjo (Ashland, OR).

### 2D-DIGE and proteomics study

Proteins were extracted from miR-1291-expressing PANC-1 cells and control cells, and subjected to 2D-DIGE and protein identification by MALDI-TOF and tandem MS (Applied Biomics, Hayward, CA), as described [57, 58]. Briefly, equal amounts of Cy3- and Cy5-labeled cellular proteins (50 μg each) were separated by 2D-DIGE, and the 2D gel images were acquired using a Typhoon Trio scanner (Amersham BioSciences, Piscataway, NJ). Scanned images were analyzed by Image Quant software (Amersham BioSciences), and a complete analysis of all differentially expressed proteins was obtained from the 2D-DIGE-derived data using Decyder software (Amersham BioSciences). Spots with differential expression and a consistent presence in replicate gels were identified and obtained using the Ettan Spot Picker. After the staining dye was removed, the samples were dried, rehydrated and digested in-gel at 37°C overnight. Digested samples were extracted, desalted, mixed with α-cyano-4-hydroxycinnamic acid matrix and subjected to MALDI-TOF MS/MS analysis (Applied Biosystems 4700 Proteomics Analyzer, Applied Biosystems, Foster City, CA). Identification of each protein spot was performed by analyzing the peptide fingerprinting MS and fragmentation MS/MS spectra, which were submitted for database search using GPS Explorer software (Applied Biosystems, Foster City, CA) equipped with the MASCOT search engine. The highest scoring hit with a protein score confidence interval more than 95% was accepted as positive protein identification for that spot.

### Pathway analysis

Proteins identified above that were altered in PANC-1 cells by miR-1291 were mapped into molecular networks using Ingenuity Pathway Analysis (IPA) (www.ingenuity.com), based on the gene's functional annotation and molecular interactions, and searched for over-represented signaling and metabolic canonical pathways and diseases.

### Luciferase report assay

The luciferase assay was performed as described previously [30, 59]. Briefly, PANC-1 cells were co-transfected with FOXA2 3′UTR luciferase reporter plasmid and miR-1291 expression or control plasmid. HepG2 cells were co-transfected with FOXA2 3′UTR luciferase expression plasmid and 50 nM miR-1291 antagomir or control oligonucleotides. Luciferase activities were determined using dual-luciferase reporter assay system (Promega, Madison, WI) and a Berthold Centro LB 960 luminometer (Berthold Technologies, Oak Ridge, TN). Relative luciferase activities were calculated as the ratio between *Renilla* luciferase and firefly luciferase and further normalized to corresponding controls.

### RNA isolation and reverse transcription quantitative real-time (RT-qPCR) analysis

Total RNAs were isolated from patient samples and human cells with Trizol reagent, and quantified using a NanoDrop spectrophotometer (Thermo Scientific, Rockford, IL). Regular and stem-loop reverse transcription of mature miR-1291 were conducted as described previously [30, 60]. RT-qPCR was performed on a MyIQ real-time PCR system (Bio-Rad, Hercules, CA). The cycle number (C_T_) at which the amplicon concentration crossed a defined threshold was determined for each individual miRNA. The relative level of each analyte over internal standard (glyceraldehyde-3-phosphate dehydrogenase, or U6) was calculated as 2^−ΔΔCT^, where ΔΔC_T_ = ΔC_T treatment group_ (analyte – internal standard) – ΔC_T control group_ (analyte – internal standard).

### Western blot

Cells were lysed in RIPA buffer (Rockland Immunochemical Inc., Limerick, PA) supplemented with complete protease inhibitors (Roche, Nutley, NJ), and protein concentrations were determined using a BCA Protein Assay Kit (Thermo Fisher Scientific Inc.). Proteins (50 μg per lane) were separated on a 10% SDS-PAGE gel and then transferred onto ECL membranes (GE Healthcare, Piscataway, NJ). Membranes were incubated with primary antibodies against AGR2 (1/10,000 dilution; Abcam, Cambridge, MA), FOXA2 (1/2,000 dilution; Abcam), or GAPDH (1/2,000 dilution, Santa Cruz Biotech Inc., Texas, TX), and then with a HRP labeled anti-mouse IgG secondary antibody (Jackson ImmunoResearch Inc., West Grove, PA). The membranes were incubated with ECL substrates for 1 min, and target proteins were visualized with a ChemiDoc XRS Imaging System (Bio-Rad, Hercules, CA).

### Immunohistochemistry and immunocytochemistry analysis

The expression of AGR2 in PANC-1 cells and PDAC patient tissues was determined by immunocytochemistry and immunohistochemistry as described before [38, 40] with modifications. Briefly, staining was done on 5-μm thick paraffin sections using rabbit anti-AGR2 antibody (Abcam, ab76473, 1:30 dilution), following protocols for the DAKO Autostainer Link 48 System. Images were captured using an Olympus camera (DP25) and CellSens software (Olympus, Center Valley, PA).

### Xenograft tumor study

All animal studies were conducted in accordance with National Institutes of Health animal use guidelines and the protocol approved by the Institutional Animal Care and Use Committee at SUNY-Buffalo. Athymic male nude (CD-1 nu/nu) mice (8 weeks old) were purchased from The Jackson Laboratory (Bar Harbor, ME). Exponentially growing control and miR-1291-expressing PANC-1 cells were harvested, resuspended in 100 μL of PBS/Matrigel (1:1) solution and injected subcutaneously (10 million cells/animal) into nude mice (12 per group). Tumor size was measured once per week for 8 weeks and tumor volume was calculated using the formula, tumor volume = 0.5 × length × width^2^. Mice were euthanized at the end of the study, and tumors were collected, weighted and subjected to the pathohistological analyses.

### Statistical analysis

All values are expressed as mean ± S.D. Different treatments (qPCR, luciferase data and tumor size) were compared by unpaired Student's t-test, one-way or two-way ANOVA with Bonferroni post-tests (Prism, GraphPad Software Inc., San Diego, CA). Difference was considered as significant if the probability was less than 0.05 (*P* < 0.05).
